# Surgery for isolated sagittal synostosis: a neuro-developmental necessity or an isolated cosmetic challenge? Mini-survey and literature review

**DOI:** 10.1007/s00381-026-07313-w

**Published:** 2026-05-14

**Authors:** Jehuda Soleman, Gianpiero Tamburrini, Jonathan Roth, Shlomi Constantini

**Affiliations:** 1 Department of Neurosurgery and Pediatric Neurosurgery, University Hospital and Children’s Hospital Basel, Basel, Switzerland; 2 Faculty of Medicine, University of Basel, Basel, Switzerland; 3 Pediatric Neurosurgery Unit, Fondazione Policlinico Universitario Agostino Gemelli IRCCS, Rome, Italy; 4 Institute of Neurosurgery, Catholic University School of Medicine, Rome, Italy; 5 Department of Pediatric Neurosurgery, Dana Children’s Hospital, Tel Aviv Medical Center, Tel Aviv-Yafo, Israel; 6 Department of Pediatric Neurosurgery, the Pediatric Brain Center, Dana Children’s Hospital, Tel Aviv Medical Center, Tel Aviv University, Tel Aviv, Israel

**Keywords:** Sagittal synostosis, Mini-survey, Cosmetic

## Abstract

**Introduction:**

While surgery of single-suture sagittal craniosynostosis (SS) is effective for cranial shape correction, its role in preventing neurocognitive impairment or intracranial pressure (ICP)–related sequelae is uncertain. We conducted a mini-survey of experts and a narrative literature review to evaluate current perspectives and evidence guiding treatment indications for SS.

**Methods:**

A mini-survey was distributed to 25 pediatric neurosurgery experts following a debate at the 2025 Varna meeting. Respondents were presented with clinical scenarios of infants with moderate or severe SS and queried regarding counseling on non-cosmetic consequences of nonoperative management. In parallel, a narrative PubMed literature review (December 2025) examined neurocognitive outcomes, ICP prevalence, treatment timing, surgical technique, and methods of ICP assessment in SS.

**Results:**

Survey responses demonstrated marked heterogeneity in expert opinion. While many surgeons viewed cosmetic correction as the primary indication for surgery, others recommended intervention to mitigate uncertain risks of neurodevelopmental delay or elevated ICP. The literature consistently shows that most children with SS achieve normal-range intellectual functioning and attend regular schools, though mild and heterogeneous vulnerabilities in language, attention, motor skills, and learning are described compared to the normal population. These differences are also detectable in infancy, suggesting a prenatal contribution to altered brain development, that may persist despite surgery. Evidence linking surgery, age at intervention, or surgical technique to improved long-term neurocognitive outcomes is inconsistent. Untreated SS carries a variable risk of elevated ICP, which is imperfectly detected by non-invasive surrogates; postoperative ICP elevation occurs in a minority of cases.

**Conclusion:**

SS is generally associated with favorable neurocognitive and developmental outcomes, though mild neurodevelopmental differences and ICP-related risks warrant careful long-term surveillance. Given limited and heterogeneous evidence, individualized counseling and shared decision-making remain essential.

**Supplementary Information:**

The online version contains supplementary material available at https://doi.org/10.1007/s00381-026-07313-w.

## Introduction

The optimal management of non-syndromic, single-suture sagittal craniosynostosis (SS) remains the subject of ongoing debate, particularly regarding whether surgical intervention should be recommended solely for cosmetic reasons or whether it may also prevent neurocognitive impairment or intracranial pressure (ICP)–related consequences. The aim of this mini-survey and literature review is to analyze expert perspectives and the current evidence regarding indications for the treatment of non-syndromic, isolated, single-suture SS.

## Methods

To explore current expert perspectives on indications for the treatment of single-suture SS, we conducted a mini-survey among 25 experts in pediatric neurosurgery triggered by the 2025 Varna meeting debate (Table 1). The experts were contacted by the senior author (SC) based on their experience in craniosynostosis surgery and their contributions to the recent literature. To minimize potential bias and promote independent interpretation of responses, the expert panel was kept anonymous. Two clinical scenarios were presented: an otherwise healthy 2-month-old infant with severe (8–10/10) SS and one with moderate (5–7/10) cranial deformity. Respondents were asked how they counsel parents regarding potential non-cosmetic consequences of nonoperative management. Survey responses were collected by email, compiled in an Excel spreadsheet, and subsequently analyzed.

**Table 1:** Results of the conducted mini-survey

Question 1: For a 2-month-old (otherwise totally normal baby) with significant classic single suture sagittal craniosynostosis with pronounced deformity (8–9-10/10), you tell the parents: The non-cosmetic results of not operating are:	
	*n* = 25
A. Almost nothing or nothing	2 (8%)
B. It is *controversial* (my opinion is…)	7 (28%)
C. The surgery has definite neuro-cognitive preventive impact and should therefore be done	11 (44%)
D. Because of the doubt, I would recommend doing it	5 (20%)
Question 2: For a 2-month-old (otherwise totally normal baby) with significant classic single suture sagittal craniosynostosis with less pronounced deformity (5–6-7/10), you tell the parents: The non-cosmetic results of not operating are:	
	*n* = 25
A. Almost nothing or nothing	3 (12%)
B. It is *controversial* (my opinion is…)	9 (36%)
C. The surgery has definite neuro-cognitive preventive impact and should therefore be done	7 (28%)
D. Because of the doubt, I would recommend doing it	6 (24%)

In addition, we performed a narrative literature review based on a PubMed search conducted in December 2025, focusing on sagittal craniosynostosis, neurocognitive and neurodevelopmental outcomes, and elevated ICP. Specifically, we aimed to assess how cognitive outcomes are influenced by treatment in patients with SS and whether untreated patients demonstrate higher rates of cognitive or developmental deficits. We further evaluated whether age at the time of treatment or surgical technique affects neurocognitive or neurodevelopmental outcomes. Finally, we reviewed the existing evidence on ICP in both untreated and treated patients with SS, examined the influence of age at treatment on ICP-related outcomes, and summarized invasive and non-invasive methods for ICP measurement described in the literature.

## Results

### Mini-survey findings

All 25 experts who were approached responded to the mini-survey. Responses revealed a wide spectrum of opinions. The survey results are presented in Table 1; comments were provided by 17 out of 25 respondents (68%) and are presented in Supplementary document 1. In cases of severe deformity, 8% of respondents believed that nonoperative management would not result in any non-cosmetic consequences, while 28% considered the issue controversial and provided additional commentary. A total of 44% recommended surgery to definitively prevent potential neurocognitive delay, whereas 20% advised surgical intervention primarily because of residual uncertainty. In cases of mild deformity, 12% of respondents felt that nonoperative management would have no impact on neurocognitive development, and 36% regarded the potential consequences as controversial. Surgery was recommended by 28% to definitively prevent neurocognitive delay, while 24% supported intervention mainly due to uncertainty regarding outcomes.

Several surgeons emphasized that current evidence does not consistently support cognitive benefits of surgery, noting that cosmetic correction remains the primary indication. They also highlighted the importance of long-term follow-up for signs of elevated ICP. Others advocated for early surgery—preferably before 6 months of age—citing the potential to support brain growth and reduce the risk of later intracranial hypertension or neurocognitive delays. Intermediate positions were also present; several respondents estimated a 10–20% risk of raised ICP in untreated cases and recommended surgery largely out of caution, given the difficulty of predicting which children might develop complications. For most responders, neither the child’s age (2 vs. 4 months) nor the severity of deformity significantly altered their recommendations. There was broad consensus that nonoperatively managed children require careful ophthalmological and developmental surveillance.

### Current evidence based on a literature review

Sagittal synostosis (SS) is generally associated with normal intellectual development and favorable functional outcomes, although subtle neurocognitive vulnerabilities are relatively common (Table 2). [1–7]. A subset of children exhibits mild deficits in domains such as language, attention, and processing speed, which may already be detectable in infancy and can persist over time despite surgical correction. [3, 8–11]. While surgery improves cranial morphology and intracranial pressure dynamics, its impact on long-term neurodevelopmental outcomes remains uncertain, partly due to the lack of untreated comparison groups [1, 12–14]. Evidence regarding optimal timing and type of surgical intervention is inconsistent, with no clear superiority established [2, 8, 11, 12, 19–21]. Importantly, most difficulties are mild and rarely functionally disabling, and overall quality of life is typically high. Nevertheless, ongoing developmental and clinical monitoring is recommended given persisting uncertainties, particularly regarding intracranial pressure and long-term cognitive trajectories [3, 22–29].

**Table 2:** Summary of neurodevelopmental and clinical features in sagittal synostosis based on the existing literature

Domain	Findings	Key details	Refs
General cognition & schooling	Mostly normal	Normal IQ; regular school attendance; milder deficits vs. other craniosynostoses	[1–7]
Subtle vulnerabilities	Present in subset	Parental concerns up to 18.5%; ADHD in substantial subset	[8]
Untreated patients	Mild deficits common	~ 40% show lower performance in learning, memory, attention; rarely disabling	[9]
Early development	Differences detectable early	Motor and early cognitive delays in infancy; possible prenatal origin (assessment challenging)	[3, 10, 11]
Long-term trajectory	Delays may persist	Motor/language delays can continue despite surgery	[1, 12–14]
Effect of surgery	Uncertain	Does not consistently normalize development; no untreated comparison studies	[1, 12–14]
Cognitive domains affected	Selective vulnerability	Language, hand–eye coordination, working memory, processing speed	[4, 15, 16]
Learning outcomes	Slightly increased risk	Reading and spelling difficulties more frequent despite average IQ	[5]
Behavioral/emotional	Generally mild	Emerge at school age with higher cognitive demands	[17, 18]
Quality of life	Favorable	Normal adaptive function; high QoL; strong parental satisfaction	[7]
Age at surgery and neurocognition	Inconsistent evidence	Some benefit of early surgery vs. no association	[8, 11, 12, 19]
Surgical technique	No clear superiority	Endoscopic, open, spring-assisted show similar outcomes	[2, 20, 21]
Intracranial pressure (ICP)	Elevated risk (untreated)	Variable prevalence depending on measurement	[22–24]
Post-surgical ICP	Generally improved	Reduced ICP, better autoregulation; recurrence in minority	[25–27]
ICP assessment	Challenging	Papilledema unreliable; invasive monitoring is gold standard	[3, 28, 29]

## Discussion

### Cognitive and neurodevelopmental outcome in untreated and treated SS

Across many available studies evaluating neurodevelopment in SS, affected children are reported to fall within the normal range of intellectual functioning, begin school at a typical age, and attend regular classes. Compared with other forms of single-suture craniosynostosis, SS is generally associated with relatively mild neurocognitive deficits [1–8]. Nevertheless, one study reported that 18.5% of parents expressed subjective concern regarding their child’s behavior or development, and nearly 40% of these children were subsequently diagnosed with attention-deficit/hyperactivity disorder [8]. In untreated SS, approximately 60% of patients demonstrate average intellectual performance, whereas around 40% perform below average in learning, memory, or attention [9]. However, long-term follow-up data are limited, and it remains unclear how many of these individuals develop persistent neurocognitive or functional impairments in adulthood, or how these early findings relate to educational attainment and employment outcomes later in life.

Several studies indicate that both non-operated and surgically treated children exhibit measurable neurocognitive vulnerabilities, although the extent and persistence of deficits vary widely. Importantly, it has been shown that despite relatively early correction of skull shape, children with SS do not achieve equivalent performance across all neurocognitive domains compared with matched controls at school age, suggesting that impairment of brain function may already occur in utero [11]. Infants with untreated single-suture synostosis demonstrate modest early developmental delays, particularly in motor development, with significantly reduced mental and motor scores prior to surgery [3, 10]. Long-term follow-up of non-operated patients at 3 and 7 years revealed minor concerns related to peer relationships and prosocial behavior at 3 years and emotional problems at 7 years, although these findings were inconsistent over time, and overall cognitive ability at 7 years was close to average [30].

While some studies report no significant neurodevelopmental differences between craniosynostosis types [10, 31], others identify significant variation, with SS associated with milder deficits and higher IQs compared with metopic and unicoronal synostosis [2]. Longitudinal studies following children after surgical reconstruction indicate that early developmental delays—particularly in motor skills—often persist, and in some cases, mental development scores decline postoperatively, suggesting that surgery alone does not normalize early neurodevelopmental differences [1, 12–14, 32–34]. Detailed analyses of mental scale components indicate that language skills as well as hand–eye coordination contribute most strongly to reduced test performance during infancy and childhood [12, 15, 16, 35]. One study identified speech, cognitive, and/or behavioral abnormalities in 39% of children with SS at 6 years of age, although surgically treated sagittal patients demonstrated lower rates of abnormalities compared with other craniosynostosis types [36]. Additionally, deficits in working memory and processing speed have been described in surgically treated SS patients [4].

Early motor delay appears to warrant particular attention, as impaired motor development in infancy has been linked to later cognitive difficulties [12, 37]. Selective neurorehabilitation programs may therefore be beneficial, with evidence suggesting improvements in visuospatial and locomotor skills by 1 year of age, whereas gains in language and learning are typically observed later, at approximately 6–7 years [35]. While many children fall within the normal IQ range, up to half demonstrate an increased incidence of learning disabilities, particularly in reading and spelling [5]. In contrast, a recently published study reported age-appropriate adaptive functioning on the Vineland Adaptive Behavior Scales, high health-related quality of life as measured by the KINDL questionnaire, and high parental satisfaction with cosmetic and neurocognitive outcomes assessed via patient-reported outcome measures [7]. Other authors observed subtle shortcomings in sustained attention and vigilance in surgically treated children, although these differences were minimal compared with normative data and unlikely to be clinically relevant [6].

Several reviews suggest that mild but persistent neuropsychological deficits may become more apparent as cognitive demands increase at school age [17, 18]. A systematic review analyzing single-suture craniosynostosis concluded that affected children are at increased risk for difficulties across language, cognitive, and motor domains both before and after surgery, without clear differences between craniosynostosis types and with limited data on modifying factors [38]. A more recent large meta-analysis found no consistent association between SS and neurocognitive delay, although attention and short-term memory difficulties were noted in some children, underscoring the importance of ongoing monitoring and support [39]. However, this meta-analysis emphasized the heterogeneity and generally low quality of available data, characterized by small sample sizes and limited use of control groups [39]. Subtle behavioral problems have also been reported in single-suture craniosynostosis overall, with SS demonstrating the lowest level of observed behavioral difficulties [40].

In summary, despite heterogeneous data and methodological limitations, a substantial proportion of children with SS exhibit mild difficulties across language, cognitive, motor, and behavioral domains. These challenges are generally not of functional significance, as most children attend regular classes and demonstrate normal-range IQ. Developmental differences are often present in infancy prior to treatment and may persist following surgical correction, supporting the hypothesis that altered brain development may begin in utero. Whether surgical intervention prevents further neurodevelopmental decline remains unclear, as direct comparisons between treated and untreated cohorts are neither ethical nor feasible. Overall, SS appears to be associated with fewer neurodevelopmental challenges than other single-suture craniosynostosis types, although the relative contribution of intrinsic and extrinsic factors remains uncertain.

### Does age at surgery impact cognitive and neurodevelopmental outcome?

The influence of age at surgical correction on long-term neurocognitive outcomes in SS remains uncertain. Some studies suggest that earlier intervention, particularly before 6 months of age, may be associated with modest improvements in specific developmental domains, including full-scale IQ and academic skills [11, 19]. However, other prospective studies found no clear association between surgical timing and subsequent mental or motor development, with postoperative developmental indices remaining below population norms regardless of age at repair [12]. In this series, the average age at surgery was approximately 9 months, limiting conclusions regarding potential benefits of surgery performed before 6 months of age [12]. A recently published large cohort study involving more than 500 surgically treated SS patients found no influence of age at surgery (5 months versus 11 months) on neurocognitive or behavioral outcomes [8]. Similarly, school-age follow-up studies have not consistently demonstrated a relationship between age at surgery and global intellectual outcomes [21]. Overall, the available evidence remains limited and inconsistent and does not conclusively support earlier surgery as a determinant of improved long-term neurodevelopment.

### Does surgical technique impact cognitive and neurodevelopmental outcome?

Comparative studies evaluating endoscopic strip craniectomy versus open cranial vault reconstruction in school-aged children report no significant differences in overall intellectual ability, with standardized test scores falling within the normative range for both techniques [21]. Subdomain analyses reveal only minor differences, such as trends toward lower working memory or processing speed in some cohorts, which are generally not clinically meaningful. Comparisons between open cranial vault surgery and spring-assisted techniques indicate that, after adjusting for covariates, open cranial vault reconstruction was associated with higher verbal, performance, and full-scale IQ, although academic achievement did not differ significantly between groups [20]. Similar findings have been reported when comparing open cranial vault reconstruction with strip craniectomy, with some authors suggesting that whole-vault cranioplasty performed before 6 months of age yields the most favorable long-term intellectual outcomes [2, 41, 42]. In contrast, other studies report no significant neurocognitive differences between surgical techniques [8], while one study suggested a potential advantage of endoscopic intervention, as social and cognitive delays were more frequently observed following open surgery [43]. Collectively, these findings indicate that although surgical technique may influence perioperative factors and cosmetic outcomes, its impact on long-term neurocognitive development remains controversial.

### Increased intracranial pressure in untreated and treated SS

Multiple studies indicate that untreated patients with isolated SS are at risk of developing elevated intracranial pressure (ICP), although reported prevalence varies widely depending on cohort characteristics and measurement methodology [23, 24, 44–47]. A recent study evaluating ICP before and after endoscopic strip craniectomy in patients with single-suture craniosynostosis suggested that the frequency and degree of ICP elevation may have been underestimated in earlier literature. In this study, SS patients demonstrated a mean ICP of 9.7 mmHg preoperatively, which decreased to 1.7 mmHg following strip craniectomy [22]. Optic disc swelling was present in 71% of SS patients prior to treatment and resolved in all cases after surgery. Overall, SS patients exhibited lower ICP values and lower rates of optic disc swelling compared with other craniosynostosis types, while also demonstrating the highest rates of postoperative improvement [22].

Comparable findings were reported by Hurth et al., who observed ICP values exceeding 15 mmHg in 77% of SS patients, with normalization to below 15 mmHg in 96% following surgical correction [25]. This study further demonstrated impaired cerebral autoregulation in patients with elevated ICP, whereas relative cerebral blood flow and velocity improved significantly after surgical repair [25]. In contrast, Wall et al. reported raised ICP in 44% of SS patients and found no reliable association between elevated ICP and clinical symptoms, developmental delay, ophthalmological findings, or radiological markers [48]. Earlier work by Thompson et al. identified raised ICP in 24% of children with SS [49]. Collectively, multiple studies over several decades confirm that untreated craniosynostosis patients are at risk of developing clinical and ophthalmological signs of raised intracranial pressure, while the rates describe are heterogeneous [23, 24, 44, 45, 47, 50].

When papilledema is used as a surrogate marker, reported rates are lower in SS, ranging from 1 to 10%, and the correlation between papilledema and elevated ICP—as well as the sensitivity of papilledema for detecting raised ICP—is low, particularly in infants and young children [29, 48, 51–53]. In contrast, in children older than 8 years, sensitivity approaches 100% [29]. This age-dependent discrepancy may be explained by greater compliance of unfused cranial sutures in infants and younger children, which may buffer the transmission of elevated ICP to the optic nerve and thereby reduce the occurrence of papilledema [29, 53]. Overall, the existing literature suggests that raised ICP occurs in a substantial proportion of patients with single-suture SS but correlates poorly with papilledema as a diagnostic surrogate. Importantly, the proportion of patients who develop clinically relevant ICP elevation leading to visual impairment, ICP-related symptoms, or cognitive dysfunction remains unknown [54]. Further, whether raised ICP in single suture craniosynostosis patients leads to neurocognitive or developmental deficits remains uncertain.

Even following cranial vault reconstruction, elevated ICP—although relatively uncommon—may persist or arise de novo in a subset of patients [26, 27, 55–58]. Studies assessing postoperative ICP using invasive monitoring, fundoscopy, or radiologic surrogates report residual or recurrent ICP elevation in 0–13% of surgically treated SS cases [7, 26, 27, 47, 55–57, 59, 60]. However, postoperative papilledema or reduced calvarial growth does not consistently correlate with elevated ICP, nor is it always clinically relevant or indicative of the need for surgical re-intervention [60]. New-onset papilledema after surgery may resolve spontaneously, while conversely, some patients with elevated ICP do not demonstrate papilledema at all [60]. Consequently, direct ICP measurement in asymptomatic patients may be warranted before considering surgical re-treatment.

### Does age at surgery impact increased intracranial pressure in SS?

The influence of age at surgical repair on the risk of persistent or secondary ICP elevation remains controversial. Some studies suggest that surgery performed before 6–9 months of age is associated with lower rates of postoperative ICP elevation [61, 62]. In contrast, other studies report higher rates of raised ICP in patients undergoing surgical correction before 6 months of age [59] or before 6–12 months of age [47, 56]. Overall, the available data suggests a possible trend toward lower rates of secondary ICP elevation with later surgical repair; however, this effect appears moderate and is likely influenced by multiple additional factors, including craniosynostosis severity, suture type, cranial morphology, genetic susceptibility, and surgical technique.

### Methods of ICP measurement in craniosynostosis

Intracranial pressure in craniosynostosis has been assessed using a variety of methodologies. Direct invasive measurement via intraparenchymal or intraventricular monitoring remains the gold standard [22, 25, 53, 63], although its use is limited by its invasive nature. Consequently, numerous non-invasive techniques, as well as clinical and radiological surrogate markers, have been explored. Fundoscopy demonstrates low sensitivity and specificity for detecting elevated ICP, as papilledema may be absent despite raised ICP, present with borderline or normal ICP values, or regress spontaneously. As a result, alternative non-invasive approaches have been investigated to improve detection accuracy.

Epicutaneous transfontanelle ICP measurement has been proposed as a reliable non-invasive technique for children younger than 1 year [64], although its applicability is limited in SS due to early fontanelle closure. More recently, devices capturing intracranial pressure waveforms via skull oscillations have been evaluated, demonstrating correlation with papilledema but not with invasive ICP measurements, thereby raising concerns regarding precision [65]. Transcranial Doppler assessment of pulsatility index has also been studied, but correlation with ICP measured via lumbar puncture was weak, and its clinical utility remains unclear [66, 67].

Optical coherence tomography (OCT) has demonstrated greater sensitivity than fundoscopy and skull growth monitoring in detecting abnormalities associated with raised ICP and skull growth arrest [68–70]. OCT showed a sensitivity of 77.3% and specificity of 95.0% for detecting ICP above 15 mmHg, and a sensitivity of 90.0% and specificity of 81.3% for ICP above 20 mmHg [3]. However, the precise role and optimal parameters of OCT in routine screening remain unclear [28]. Optic nerve sheath diameter (ONSD) assessed on low-dose CT or MRI has also been evaluated, demonstrating a sensitivity of 65% and specificity of 64% for detecting ICP ≥ 15 mmHg [71, 72]. Ultrasound-based ONSD measurement, with or without correlation to deformability indices, has been proposed as a bedside, radiation-free alternative, although large validation cohorts are lacking [73–75].

Additional non-invasive approaches, including venous ophthalmodynamometry, acoustic elasticity, tympanic membrane displacement, and assessment of cerebral metabolic changes using near-infrared spectroscopy, have been described, but their relevance in craniosynostosis remains uncertain [76]. Neuroimaging markers such as white matter apparent diffusion coefficient changes during the cardiac cycle, sulcal effacement, basal cistern effacement, and alterations in cranial bone thickness and density have also been proposed as indicators of raised ICP, although their diagnostic value in craniosynostosis is not yet established [76–78]. Clinical surrogates continue to play an important role in patient assessment, but they are often nonspecific and may occur in the absence of elevated ICP. In summary, invasive ICP monitoring remains the most accurate method, whereas non-invasive techniques and surrogate markers offer safer alternatives but are limited by variable sensitivity and specificity.

### Interpretation and outlook of our mini-survey and literature review

The mini-survey presented underscores the diversity of expert opinion and highlights the ongoing uncertainty surrounding the neurodevelopmental implications of untreated SS. Until high-quality prospective multicenter data become available, the management of SS will continue to balance cosmetic, developmental, and psychosocial considerations. Shared decision-making with families and vigilant long-term surveillance remain cornerstones of care. These findings should encourage further collaborative research to clarify the developmental trajectory of children with SS and develop methods for non-invasive ICP measurement with high sensitivity which will help to refine surgical indications.

## Conclusion

Non-syndromic, single-suture SS is generally associated with normal intellectual functioning, although mild and heterogeneous neurodevelopmental vulnerabilities are common and may be present before and after treatment. The mini-survey revealed substantial variability in expert opinion, with most surgeons viewing cosmetic correction as the primary indication for surgery, while others recommend early intervention out of concern for potential intracranial pressure– or development-related risks. Current evidence does not conclusively demonstrate that surgery prevents neurocognitive decline, nor does it clearly establish age at surgery or surgical technique as determinants of long-term developmental outcome. Untreated patients carry a variable risk of elevated intracranial pressure, and postoperative ICP elevation may persist in a minority of cases, further complicating management decisions. In the absence of definitive comparative data, individualized counseling, shared decision-making, and vigilant long-term surveillance is recommended.

## Supplementary Information

Below is the link to the electronic supplementary material.

ESM 1 (DOCX.17.9 KB)

## Data Availability

No datasets were generated or analysed during the current study.
