# Supplementary material for: Surgery for isolated sagittal synostosis: a neuro-developmental necessity or an isolated cosmetic challenge? Mini-survey and literature review
Source: Childs Nerv Syst. 2026 May 14;42(1):218. doi: 10.1007/s00381-026-07313-w (PMC13652866; doi:10.1007/s00381-026-07313-w)
Supplement: Supplementary file 1 [file 381_2026_7313_MOESM1_ESM.docx]

**Supplementary document 1**

Comments from the responders

“I would never push a family towards surgery because of potential neuro issues (including the issue of late ICP)”

“But perhaps high ICP along the way? “

“For a two-month-old or four months old otherwise totally normal the thoughts are the same… Yes, I recommend surgery due to doubts about the neurocognitive and visual future, although unlikely”

“I tell them there is very little or consistent data on cognitive outcomes and surgery for scaphocephaly whether it is done via endoscopic or open technique. 
We do have data on kids presenting at older ages (> 2.5 yrs) who do manifest increased ICP (and anecdotal evidence that their cognition improved after calvarial expansion) but no one knows what the denominator is. Surgery at this age is strictly for normalization of the calvarial shape which would benefit eventual psychosocial development and if the patient is less than 5-5.5 months old.“

“Almost nothing or nothing (independent of less or more pronounced deformity) – I would say that there is a 10-20% chance of elevated ICP, based on the literature (and my experience when families have opted not to have surgery).  The neurocognitive impact of not operating is uncertain in any given child and the primary purpose of surgery is cosmesis.  I then leave the decision to the family and follow the nonoperative kids closely for papilledema or other signs of increased ICP.  I follow the operative kids for this as well, somewhat less closely, since there is a 5% risk of increased ICP/Chiari/headaches in this group as well, as a result of suture reclosure or closure of other sutures, since a small subset has an unrecognized syndrome.”

“I based my ballpark estimate on the results from the Necker group, which were published, and also my own personal experience from about 400 cases of sagittal synostosis. We did publish the results of a “late onset” cohort - kids who were missed initially. It’s definitely more of an issue than pure cosmesis, but in any given child, it is impossible to predict, so I emphasize the cosmetic rationale for surgery, and mention the ICP aspects in passing. I never use that as the reason for surgery.”

“I usually say that sometimes without surgery the child may develop increased intracranial pressure and may need cranial expansion”

“The risk of raised ICP is significantly lower in children submitted to surgery compared to those who are not. No change if 2 months or 4 months. No change if mild or severe in less than 12 months.”

“In principle, the indication for surgery is mainly based on cosmetic factors. However, we do know from the literature and own experience that some patients will develop increased intracranial pressure over time, especially in severely effected infants. That might cause chronic headaches and might also have consequences of functional outcome. We, however, don’t know what is the proportion of patients who will develop headaches and if the individual case will be affected by it in the future. That is what I tell the parents.”

“I tell the parents there is a risk of 15% for related cognitive delay and that surgery, especially if early, is protective against this risk.”

“Although controversial, in these cases I suggest surgery, considering potential neuro-cognitive, seizures, Chiari I and other manifestations. Unfortunately, in our country some cases al diagnosed on very late situations, after 2-4 y-o. And in those we sometimes struggle with follow-up and awareness of potential neurological deficits.”

“I explain that most children without treatment have a normal brain, but that the literature suggests approximately 15% risk of developmental problems if untreated (for years). Same for 2- or 4-month-old child.”

“I do not promise any improvement in neurocognitive or speech and language delay. Quote some observations of rapid improvements noted in a few cases.”

“The risk of intracranial hypertension is around 10% in scaphocephaly (4-14%). It’s not possible to identify which patient will develop high ICP with our resources (MRI/perfusion study). The early diagnosis and early surgery (<6 months) are very important in order to reduce the risk to develop neurocognitive delay/ difficulties. The risk of intracranial hypertension is not correlate to the severity of the deformity and we cannot assure of the absence of consequences on the neuro-cognitive development without surgery. 
Because the baby is in the perfect period to plan the surgery with the highest possibility to reduce the risk of consequences in the future, the surgery is always indicated.”

“In addition to a potential psychosocial disadvantage of having a head deformity, I quote that depending on the literature between 8-30% could suffer from elevated ICP in childhood necessitating a much more complex surgery in the future. If the child is 2 or 4 months old would not change my attitude. If the child is 1.5 - 2 years old I would change my attitude and wait and watch, do periodic neurological and ophthalmology exams.“

“Early treatment helps in brain growth and prevents neurocognitive deficits ( language/learning disabilities)”

“The surgery has definite neuro-cognitive preventive impact and should therefore be done. The answer remains the same for all ages”

“I am a big advocate of allowing full growth potential for the brain and would recommend surgery at 2 months of age in order to proceed with early correction. This would not change at 4 months of age. Given my previous stance I don’t believe that severity has been related to the neurocognitive issues and therefore recommend surgery, but would not push if the parents are satisfied with the mild deformation at either age and would recommend close follow up of cognitive milestones. “

 “The surgery is always indicated.”
